# New molecular and macroscopic understandings of novel green chemicals based on Xanthan Gum and bio-surfactants for enhanced oil recovery

**DOI:** 10.1038/s41598-024-63244-z

**Published:** 2024-06-03

**Authors:** Arezoo Rezaei, Saeed Karami, Amir Mohammad Karimi, Hamid Vatanparast, Saeid Sadeghnejad

**Affiliations:** 1 Department of Petroleum Engineering, School of Petroleum Engineering, Amirkabir University of Technology, Tehran, Iran; 2 Research and Development Division, Petro Atlas Zagros, Tehran, Iran; 3 Petroleum Engineering Research Division, Research Institute of Petroleum Industry (RIPI), Tehran, Iran; 4 Department of Petroleum Engineering, Faculty of Chemical Engineering, Tarbiat Modares University, Tehran, Iran

**Keywords:** Molecular characterization, Microfluidic Injections, Leaf-derived surfactants, Xanthan Gum, Asphaltene, Engineering, Chemical engineering, Environmental sciences

## Abstract

This research investigates the interactions between a novel environmentally friendly chemical fluid consisting of Xanthan gum and bio-based surfactants, and crude oil. The surfactants, derived from various leaves using the spray drying technique, were characterized using Fourier-transform infrared (FTIR) spectroscopy, zeta potential analysis, Dynamic light scattering, and evaluation of critical micelle concentration. Static emulsion tests were conducted to explore the emulsification between crude oil and the polymer-surfactant solution. Analysis of the bulk oil FTIR spectra revealed that saturated hydrocarbons and light aromatic hydrocarbons exhibited a higher tendency to adsorb onto the emulsion phase. Furthermore, the increased presence of polar hydrocarbons in emulsion phases generated by polar surfactants confirmed the activation of electrostatic forces in fluid–fluid interactions. Nuclear magnetic resonance spectroscopy showed that the xanthan solution without surfactants had a greater potential to adsorb asphaltenes with highly fused aromatic rings, while the presence of bio-based surfactants reduced the solution's ability to adsorb asphaltenes with larger cores. Microfluidic tests demonstrated that incorporating surfactants derived from Morus nigra and Aloevera leaves into the xanthan solution enhanced oil recovery. While injection of the xanthan solution resulted in a 49.8% recovery rate, the addition of Morus nigra and Aloevera leaf-derived surfactants to the xanthan solution increased oil recovery to 58.1% and 55.8%, respectively.

## Introduction

Despite global efforts to diversify energy sources beyond petroleum reserves, oil and gas remain a predominant energy source. As oil reservoirs are depleted through continuous production, new methods are being employed to counteract the loss of reservoir pressure. These methods, which alter the reservoir's chemophysical properties to enhance oil recovery, are collectively known as Enhanced Oil Recovery (EOR). Chemical methods used in EOR include polymer, surfactant, alkaline, ion-tuned water, and combinations of these chemicals. Among these methods, polymer-surfactant injection is widely utilized in EOR. Polymers are added to the injection fluid to increase viscosity, thereby improving mobility control and mitigating fingering phenomena. Surfactants, on the other hand, function through mechanisms such as interfacial reduction, wettability alteration, and emulsification^1–6^. When considering the challenges associated with surfactant and polymer injection, such as retention and adsorption, these chemicals are carefully utilized in formulating the injection fluid. However, fluctuations in oil prices can limit the selection of economically viable polymers and surfactants suitable for field injections^7^. In this regard, using natural polymers and surfactants seems to have a promising future in the oil industry.

The main limitations of using xanthan gum for improving oil production are low thermal and salinity stability at the reservoir condition^8–10^. Despite the limitations related to xanthan gum, the effectiveness of this natural polymer in enhancing oil recovery has been well-established^11^. Fu et al. 2022 used an etherification reaction to implement long aliphatic chains on the xanthan structure, which led to the improvement of rheological behavior, temperature, and salinity resistance. In the coreflooding tests, the recovery factor of modified xanthan gum injection was 7% higher than the injection of ordinary xanthan gum^12^. Nazarahari et al. 2022 used a new nanocomposite including SiO_2_, Montmorilant, and Xanthan gum to improve oil recovery. After characterizing the nanocomposite through standard tests, such as FTIR, TGA, and XRD, the wettability alteration, interfacial tension, and coreflooding tests were conducted at various nanocomposite concentrations. At the optimum concentration, the reduction of interfacial tension (56%) and rock hydrophobicity (78%) resulted in an improved oil recovery of 11.72%^13^. Xu et al. 2023 synthesized the Amide- and alkyl-modified nanosilicas to improve the salinity and temperature tolerance of xanthan gum. In the sandpack flooding tests at reservoir conditions, the injection of xanthan gum and modified nanomaterials hybrid led to the production of 11.3% more than xanthan gum injection^14^. From an atomistic insight, Luan et al. 2023 investigated CO_2_ storage using molecular dynamics simulation. Based on their simulation, the CO_2_ storage strongly depends on the dead-end pore sizes. Larger pores are harder to be filled with CO_2_ molecules, while the smaller ones have a higher efficiency. They also showed that the injection pressure strongly influences the storage; increasing the pressure difference leads to the entrance of CO_2_ molecules to the dead-end pores, enhancing the sequestration^15^.

The natural surfactants were also added to the xanthan solution to improve the surfactant-polymer performance for oil production. Machale et al. 2019 synthesized natural surfactants using Water hyacinth to improve the thermal stability and rheological behavior of the xanthan gum. Based on the TGA analysis of the synthetic surfactant, 96% of the surfactant mass was retained at the temperature of 385 K, indicating the high thermal stability of the surfactant. Besides, the xanthan gum in the presence of synthetic surfactant showed higher viscosity (with respect to the xanthan solution) in various shear rates, which is an improvement for the xanthan rheological behavior^16^. Navaie et al. 2022 added Chuback-derived surfactant to a xanthan gum solution to improve the solution performance. Based on contact angle measurements, the xanthan-Chubak-derived surfactant could improve the wettability toward oil wetness by 70% and 82% in carbonate and sandstone rocks, respectively^17^. Saha et al. 2019 used Reetha-extracted surfactant synergic with the xanthan gum and nano-silica to improve oil recovery. The additional oil recovery because of injection of composite fluid, including xanthan gum, nano silica, and Reetha-extracted surfactant, was attributed to the ability of composite solution for interfacial reduction, wettability alteration, rheology improvement, and the ability for emulsification^18^.

Naturally derived surfactants have been extensively used for improving oil production. Regarding the fact that natural surfactants are environmentally friendly and economically feasible, they have gained attraction in recent years. The efficiency of natural surfactants for wettability alteration, interfacial reduction, shale hydration inhibition, and foam generation has been proved extensively^19–27^. These natural surfactants are saponins extracted from the leaves of Morus nigra, Zyziphus spina christi, Trigonella foenum-graceum, Seidlitzia rosmarinus, Glycyrrhiza glabra, and Tragacanth, Henna trees. The saponins are natural compounds in the cells of the plant leaf. Due to their amphiphilic structure (containing triterpenes and glycoside groups), the saponin could behave as a surface active molecule in the fluid interface^28,29^. Regarding the fact that saponins are surface active molecules, which are derived from plant leaves, they could be considered as a bio-based surfactant.

The fluctuation in oil prices highly restricts employing chemical enhanced oil recovery, such as polymer and surfactant flooding. While polymer-surfactant flooding has its unique mechanisms for improving oil recovery, employing the method is challenging at low oil prices. Hence, a new economic formulation is highly required to be used as a substitution for costly synthetic polymer-surfactant flooding. This research introduces a new natural polymer-surfactant composite fluid comprised of xanthan polymer and bio-based surfactant extracted from leaf wastes. Because both of the chemicals are natural, and even the surfactants are extracted from the waste, they are more economical than synthetic materials. Although many researchers have addressed the feasibility of the xanthan gum solution, the hybrid of the xanthan with a bio-based surfactant solution still is poorly understood. Besides, the studies of the xanthan composite solution have been limited to investigating its performance through macroscopic tests, such as interfacial tension. This study shed light on the fluid–fluid behavior of xanthan-bio-based surfactant and oil from practical and molecular aspects. The emulsification, viscosity alteration, and microfluidic injection were used to probe all aspects of fluid–fluid interactions. To have a molecular insight, the emulsification of xanthan-bio-based surfactants was analyzed through Fourier-transform infrared (FTIR) spectroscopy, Nuclear magnetic resonance (NMR), and oil phase viscosity. The viscosity measurement was used to study the macroscopic effects of emulsification on the oil phase. In addition, microfluidic injections were employed to investigate the fluid–fluid behavior from a dynamic point of view.

## Materials and methods

The emulsification, rheological behavior, and microfluidic displacement of the natural surfactant-polymer solution were studied using analytical techniques (FTIR and NMR), viscosity measurement, and micromodel injections. The employed materials and techniques are discussed as follows.

### Materials

#### Crude oil

The crude oil, which was sampled from an oil field in the southern of Iran was used. The Saturate, Aromatic, Resin, and Asphaltene (SARA) contents of the crude oil are 48.2, 25.7, 17.6, and 8.5 wt.%, respectively. The crude oil can be considered as a heavy oil sample (API = 20.48 ^∘^) with a high content of organic acids (Acid number = 3.28 mg KOH/g oil).

### Natural surfactant-polymer solution

#### Natural surfactant preparation

The natural surfactants were extracted from the leaf of Morus nigra, Aloevera leaf, Zyziphus spina christi, and Glycyrrhiza glabra using the spray drying technique. The leaves were collected from the waste of trees in December. After the collection of leaves, they were dried at 90 °C for 5 days, leading to vaporizing the water and volatile organic compounds. The dried leaf was crushed into fine powders and added to distilled water at a concentration of 10 g/L. The resultant mixture was kept at 90 °C for 10 days. The resultant water, which is now turned into a dark color, contains the water-soluble parts of the associated leaf. After filtration of the dark solution by the Whatman filter paper (#42), the dark solution without fine particles was filtrated. After vaporizing the distilled water, the surfactant was obtained. Because the surfactants were extracted in the lab, it was required to characterize their properties. Hence, the FTIR, CMC calculation, and zeta potential measurements were conducted to characterize the naturally extracted surfactants.

#### Xanthan polymer preparation

The Xanthan gum, which is one of the natural polymers employed for improving oil recovery, was purchased from Sigmachemical Company.

### Methods

#### Surfactant characterization

Regarding the fact that the bio-based surfactants were extracted from the leaf, they needed to be characterized using analytical tests. To study the chemistry of the extracted surfactants, FTIR spectroscopy was employed. The FTIR spectra were captured by a refractometer (Bruker, USA) in the absorbance version, facilitating the use of integral areas. The spectra were obtained in the range of 400–4000 cm^−1^ with a resolution of 1 cm^−1^. After the identification of the FTIR peaks, the area below each peak was employed to calculate the computational data from the FTIR spectra. Hence, using the FTIR spectra, the aromaticity, aliphaticity, polarity, and carbonyl content of the surfactants were determined. The zeta potential measurements were used to study the surface electric charge of the surfactant solutions. Regarding the fact that the surfactants had an ionic nature, to investigate the critical micelle concentration (CMC) of the leaf-derived surfactants, the conductivity measurement was employed. In this regard, the extracted bio-based surfactants were added to distilled water to prepare various solutions in the concentration range of 0–50 g/L. After identifying the slope changes in the data sets of electric conductivity, the interception of two lines was reported as the CMC point. The conductivity measurements were conducted by an electrical conductivity meter (Hanna, USA) at the ambient temperature.

#### Static emulsion tests

The static bottle tests were used to investigate the emulsification behavior of the natural polymer-surfactant solutions. To do so, an identical volume of the oil and solution samples (20 ml) was mixed in a bottle tube. After mixing the oil and polymer-surfactant solution in the bottle, it was sonicated in an ultrasonic bath with a radiation power of 800 watts at the wave frequency of 37 kHz for 30 min. Then, the bottles were sustained vertically at 90 °C for 20 days. At this stage, the initial solutions were turned into three phases: aqueous solutions, emulsion, and bulk oil from bottom to top. The important part of gathering information from the emulsion tests was sampling from the bottle tests. Because there is no visually detectable margin between the oil and emulsion phases, the bulk oil phase was sampled. It should be mentioned that the emulsion stabilizing is a continuous process, and sampling from the bulk phase should be conducted at a suitable time. The proper time for sampling (20 days) was determined using trial and error tests.

The polymer-surfactant solutions were provided at two surfactant concentrations of 0.2 g/L and 0.5 g/L and two polymer concentrations of 2 g/L and 5 g/L. The emulsification of the solutions was studied by investigating the bulk oil chemistry and asphaltene molecular structures.

#### NMR spectroscopy

The NMR spectroscopy was investigated to address the molecular structure of asphaltene. The asphaltenes of the bulk phase were extracted using the standard method of the IP-143. The oil sample was mixed with normal heptane (Merck, Germany) with a volumetric ratio of 1:30. To enhance the mixing, the oil-heptane mixture was heated and stirred for 30 min at 80 °C. Afterward, the mixture was sustained away from the light for 24 h. Afterward, the mixture was filtered by a Whatman paper (#42) to separate the solid precipitations, including asphaltenes and waxes. Then, the contents of the Whatman paper were washed with normal heptane to remove the non-asphaltenes solids using a soxhlet apparatus. Afterward, the Whatman paper was refluxed using toluene to dissolve the asphaltenes. Finally, after the vaporizing of toluene, the asphaltene powders were obtained.

As mentioned before, the molecular structure of the extracted bulk asphaltene needed to be addressed using the NMR technique. In this regard, the asphaltene powders were mixed with deuterated chloroform to prepare the sample for Proton nuclear magnetic resonance (HNMR) and carbon nuclear magnetic resonance (CNMR) at the concentrations of 5 mg/0.7 ml and 40 mg/0.7 ml, respectively. A Bruker spectrometer (USA) was employed to capture the NMR spectra at the wave frequency of 500 MHz.

The y-axis in the NMR spectra (both HNMR and CNMR) is normalized intensity in a way that the respective integral area below a peak has a direct relationship with its concentration in the material. The x-axis indicates the chemical shift in terms of ppm, and it is used to identify a specific functional group or hydrogen/carbon molecular environment. Regarding the fact that the normalized integral area of a peak in NMR spectra is directly proportional to the corresponding functional group or bond, normalized integral areas were used to elucidate the structural parameters. The structural parameters of aromaticity, shape factor, carbonyl index, and number of fused aromatic rings were obtained using CNMR spectra. Besides, the length of associated aliphatic chains in terms of carbon numbers was calculated from HNMR spectra using the Dickson equation. Regarding the fact that asphaltenes are polydispersed molecules, the structural parameters obtained from the NMR spectra represent an average value for the asphaltenes^30^.

#### FTIR spectroscopy

While the nuclear magnetic resonance (NMR) methodology can yield insights into the structural characteristics of asphaltene, it may exhibit limitations in accurately discerning non-hydrocarbon-based functional groups, such as sulfoxide groups, within the NMR spectrum. To study the functional groups and chemistry of the asphaltene fraction bulk asphaltene, the FTIR technique was employed. In this context, the asphaltene powders were combined with potassium bromide (KBr) at a proportion of 1:40. A refractometer (Bruker, USA) was utilized to acquire FTIR spectra within the wavelength range of 400–4000 cm^−1^, employing a resolution of 1 cm^−1^. To obtain numerical data from the FTIR technique, the FTIR spectra were recorded in the absorbance mode. The integral areas from the start to the end of each peak were calculated to obtain the characteristic indexes reported by Karami et al.2022^31^. Using the characteristic equations, the sulfoxide, polar, carbonyl, Aromatic/ Aliphatic, and condensing indexes were obtained.

#### Viscometric analysis

It is widely recognized that the emulsification of crude oil and aqueous solutions results in the adsorption of amphiphilic hydrocarbons into the emulsion phase, potentially inducing alterations in the chemistry of the oil phase. Because the oil viscosity is a function of comprising hydrocarbon, the alteration of oil phase chemistry changes the oil phase viscosity. Hence, the rheological behavior of the oil sample could act as a tool to address the changes of dispersing and dispersed hydrocarbons. To conduct the viscometric tests, the oil sample was collected from the bulk oil phase of the emulsion. The viscometric tests were conducted by a Brookfield viscometer (USA). It should be mentioned that the repeatability of viscometric tests was $\pm$ 0.3 cP.

#### Microfluidic tests

The fluid flow in the porous medium is strongly controlled by fluid–fluid interactions (such as emulsification and interfacial tension) and rock-fluid (wettability)^32,33^. To study the fluid–fluid behavior in a dynamic displacement test, microfluidic tests were conducted. A random porous medium was designed using AutoCAD software. The pattern was sketched on the glass using a laser printing technique. After polishing the printed glass, two lines were drilled to provide paths for fluid injection. The printed glass was fixed on another glass, and placed in a furnace to be fused. The temperature of the furnace was increased to 850 °C step by step, and then, reduced to room temperature for 24 h. After placing two paths for injecting fluids, the micromodel was finally prepared for microfluidic tests.

To conduct the microfluidic tests, the micromodel was initially saturated with crude oil. The injecting fluid was injected into the saturated micromodel with an injection rate of 0.05 ml/min. A Canon camera (EOS 2000D), which was connected to a data-gathering system, was employed to monitor the fluid displacement in the micromodel. It is worth noting that after three repeated injections, the microfluidic tests exhibited a repeatability of ± 0.5%.

## Results and discussions

### Surfactant characterization

#### Surfactant FTIR

**Figure 1:**
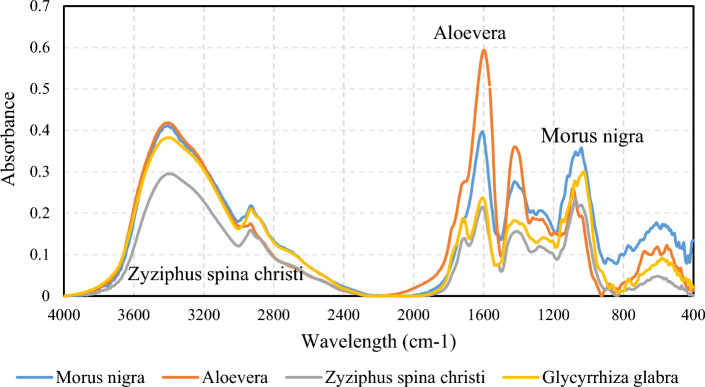
The FTIR spectra of the leaf-derived surfactants.

Regarding the fact that the chemistry of extracted surfactants could be affected by climate and the season of the leaf collection, individual surfactants need to be characterized. The FTIR technique was employed to address the functional groups present in the leaf-derived surfactants. Figure 1 illustrates the FTIR spectra of the surfactants recorded in absorbance mode.

According to the FTIR spectra, the leaf-derived surfactants have similar functional groups with different intensities. The aromatic C=C and C–H bonds are vibrating at the ranges of 1506–1680 cm^−1^ and 2820–3000 cm^−1^, respectively. The polar functional groups, such as O–H and N–H bonds, vibrate at the range of 3100–3600 cm^−1^. The carbonyl-based functional groups, such as aldehydes, carboxylic acids, and esters, could be captured at 1680–1800 cm^−1^, representing C=O bonds. In addition, the Alkyl halide and C–O bonds are represented by the peaks at the ranges of 460–590 cm^−1^ and 950–1136 cm^−1^, respectively.

To find the relative content of each functional group, the integral area of each peak was divided by the total areas (Table 1). In this regard, the total, aliphatic, aromatic, carbonyl, and halide indexes were calculated to address the surfactant chemistry. The hydrophobic part of the surfactants are generally aliphatic and aromatic rings (such as aglycone)^34^. According to the aliphatic and aromatic indexes, the Zyziphus spina christi and Aloevera-derived surfactants respectively have the highest and lowest hydrophobic parts. The polar indexes, representing O–H and N–H contents, are almost identical in all leaf-derived surfactants. The carbonyl index of Aloevera and Glycyrrhiza glabra leaf-derived surfactants was higher than other ones. In addition, the concentration of the Halide functional groups was higher in the Morus nigra and Aloevera surfactants. Based on the FTIR characteristic indexes, the functional groups with more polarity, such as carbonyl and halide, are higher in Aloevera and Morus nigra surfactants, indicating more hydrophilicity. In contrast, considering the higher aliphatic indexes of the Zyziphus spina christi and Glycyrrhiza glabra-derived surfactants, the hydrophobic part of the mentioned surfactants is higher. Based on the FTIR peak, while the overall structures and backbone of all bio-based surfactants are similar, there are some differences in functional group contents. It will be shown that some slight differences in bio-based surfactant structure lead to considerable changes to surfactant behavior.

**Table 1:** The characteristic indexes of the leaf-derived surfactants given by FTIR spectra.

Indexes	Morus nigra	Aloevera	Zyziphus spina christi	Glycyrrhiza glabra
Polar	0.411	0.426	0.455	0.425
Aliphatic	0.073	0.064	0.092	0.085
Aromatic	0.101	0.179	0.105	0.074
C=O	0.026	0.048	0.034	0.043
Halide	0.079	0.05	0.035	0.024

#### Zeta potential

**Figure 2:**
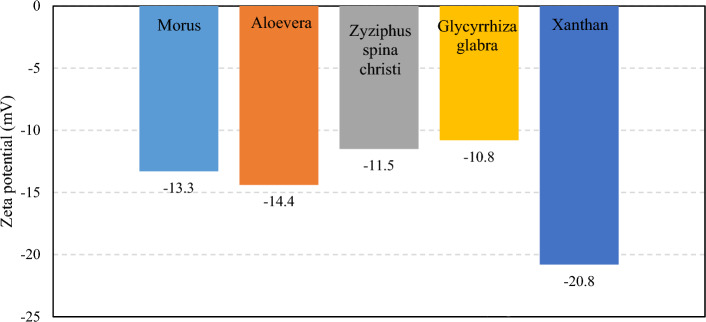
The zeta potential of the xanthan (2 g/L) and xanthan-surfactant (400 ppm) solutions.

Regarding the fact that electrostatic interaction between the surfactant and hydrocarbon plays a vital role in the emulsification, the surface charge of the surfactants needs to be determined. The zeta potential was measured to study the surface charge of the dissolved surfactants. The zeta potentials of the surfactant solutions (at a concentration of 400 ppm) are depicted in Fig. 2. As shown in Fig. 2, the zeta potential of the xanthan gum solution with a concentration of 2 g/L is -20.8 mV, which reduces to the range of -10.8 to -14.8 mV after the addition of leaf-derived surfactants. This observation indicates that the presence of the surfactants suppresses the surface electric field of the xanthan polymers. Among the surfactants, the ones extracted from the Glycyrrhiza glabra and Aloevera leaves have the lowest (-10.8 mV) and highest (-14.4 mV) zeta potential values, respectively. Hence, it could be deduced that the highest and lowest stability among the surfactants is observed in the ones extracted from Aloevera and Glycyrrhiza glabra leaf waste, respectively. The trend of the zeta potential is consistent with the aromatic index, indicating that the higher aromatic content of the bio-based surfactant leads to higher stability.

#### DLS

**Figure 3:**
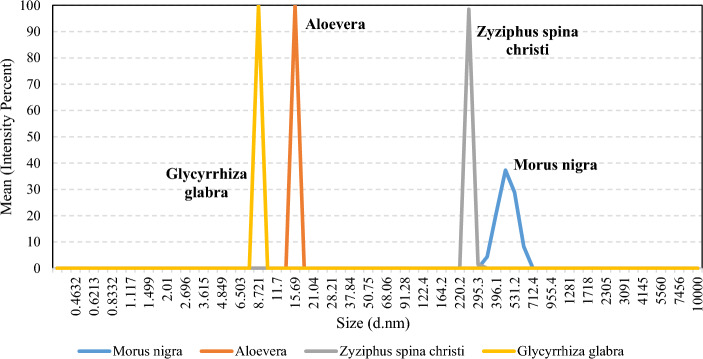
The DLS spectra of the leaf-derived surfactants at the concentration of 0.2 g/L in xanthan gum with the concentration of 2 g/L.

The Dynamic light scattering (DLS) technique was conducted to obtain the size distribution of surfactant aggregates. As shown in Fig. 3, the average diameter of the Morus nigra, Aloevera, Zyziphus spina christi, and Glycyrrhiza glabra surfactants are 459.3, 15.77, 256.9, and 8.768 nm, respectively. Regarding the DLS spectra, the surfactant aggregates are the smallest in Glycyrrhiza glabra and Aloevera solutions. The trend of DLS changes is almost consistent with the changes in the C=O index. Higher C=O-based functional groups (ketones, carbonyls, carboxylic acids, and aldehydes) probably lead to higher potential for aggregations. This observation could be also explained by the Brønsted acid–base theory. The carboxylic acids and O–H-based functional groups could act as hydrogen donors and acceptors, providing the intermolecular force for aggregation. The smaller aggregates, which lead to more surface area, increase the chance for hydrocarbon-surfactant interaction.

#### CMC measurement

**Figure 4:**
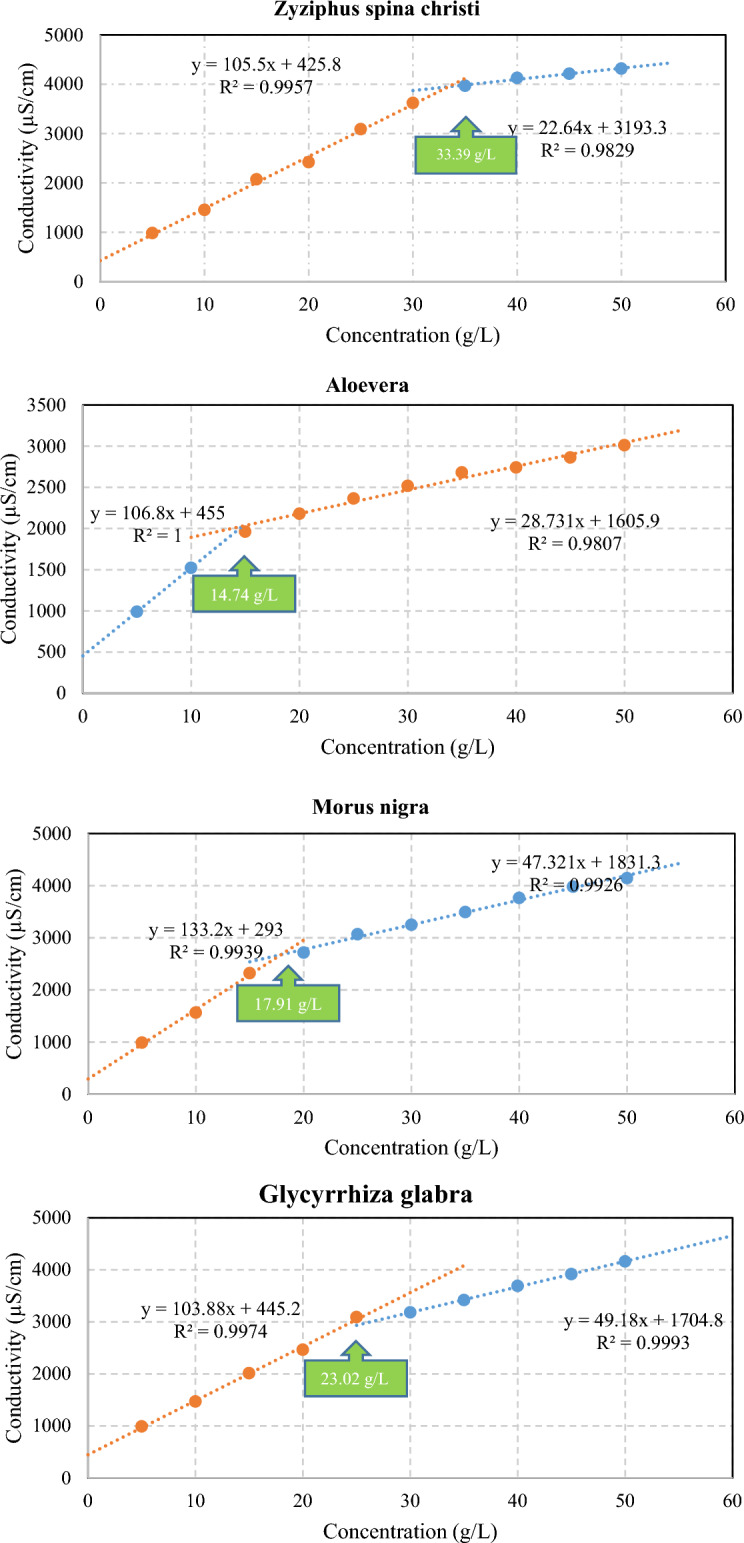
The CMC calculations obtained from the intersection of two trend lines caused by graph (electric conductivity vs concentration) slope change.

The critical micelle concentration (CMC) of surfactants was determined using electrical conductivity measurements. The electric conductivities of the various bio-based surfactant solutions versus concentration are shown in Fig. 4. Based on the conductivity measurement tests, the CMC of the surfactants extracted from the Zyziphus spina christi, Morus nigra, Glycyrrhiza glabra, and Aloevera were 33.39, 17.91, 23.02, and 14.74 g/L, respectively. It should be mentioned that the obtained CMC is consistent with the range of reported data in the literature review^21,35^. According to the CMC calculation tests, the Aloevera-derived surfactants, which have the lowest CMC, are more feasible in less concentration for generating micelles. Due to the electrical nature of bio-based surfactants, the electrical conductivity method was employed to measure the CMC. The CMC of bio-based surfactants is directly proportional to the aliphatic index, given by the FTIR technique. In other words, higher aliphaticity, as an indication of surfactant hydrophobic content, resulted in higher CMC.

#### Bulk oil FTIR

**Figure 5:**
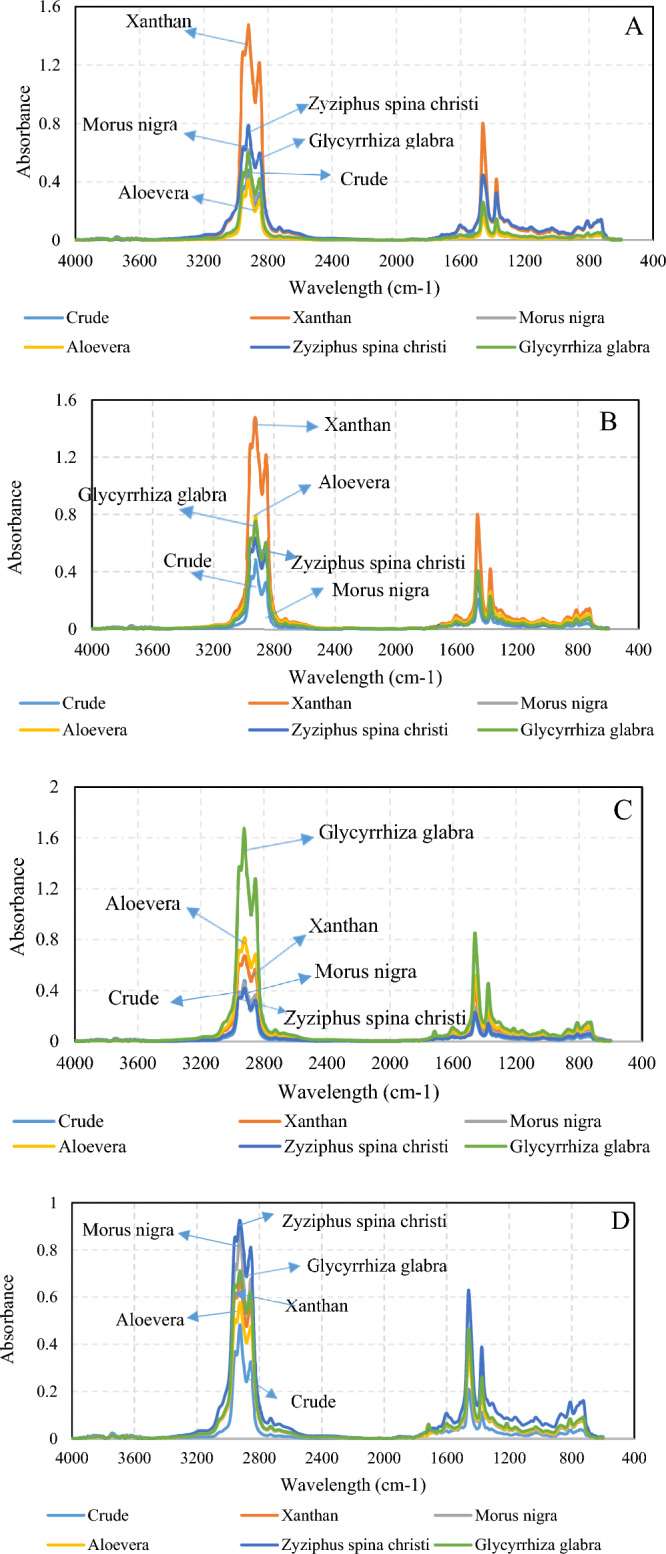
The FTIR spectra of the oil phases of emulsions generated by (**A**) xanthan (2 g/L)-surfactant (0.2 g/L), (**B**) xanthan (2 g/L)-surfactant (0.5 g/L), (**C**) xanthan (5 g/L)-surfactant (0.2 g/L), and (**D**) xanthan (5 g/L)-surfactant (0.5 g/L).

It has been extensively demonstrated that the emulsification of crude oil and a surfactant solution, generates three phases comprising bulk oil, an emulsion phase, and a surfactant solution phase. Because the separation border of the emulsion phase cannot be identified from a technical point of view, the properties of the oil bulk phase are investigated to address the affinity of compounds toward the emulsion phase. In this regard, the FTIR of the bulk phase is studied to address the hydrocarbon-surfactant interaction in the emulsion phase. Based on the identified peaks in the FTIR spectra, the oil phase has aromatic, aliphatic compounds, as well as functional groups, such as amine, alcohols, sulfoxide, and carbonyl compounds^31,36–42^. The FTIR indexes were measured using equations suggested by Karami et al. 2022^31^. Figure 5 shows the FTIR spectra of the bulk oil phases, which were in contact with xanthan gum solution with different surfactants.

As shown in Table 2, the Aromatic/Aliphatic indexes of the oil phases in contact with xanthan and xanthan-surfactant solutions (0.06–0.186) were generally higher than that in crude oil (0.06). Hence, the aliphatic hydrocarbons have more affinity to participate in the emulsion phase. The higher Aromatic/Aliphatic index of the oil sample in contact with Zyziphus spina christi (0.186) represents a lower ability of this surfactant to interact with the aromatic hydrocarbons. A higher condensing index value for the oil phase, in comparison to crude oil, signifies a diminished affinity of polyaromatic hydrocarbons towards the emulsion phase. In other words, in comparison to fused aromatic hydrocarbons, lighter aromatic hydrocarbons have a greater contribution to the emulsion phase. According to the indexes of condensing and Aromatic/Aliphatic, the Zyziphus spina christi and Morus nigra respectively have the lowest and highest interaction with aromatic hydrocarbons. The only oil phase that has a lower aliphatic length index is the one in contact with the Zyziphus spina christi polymer solution (1.360). Hence, except for the Zyziphus spina christi, the lighter saturated hydrocarbons have more tendency to participate in the emulsion phase. This observation aligns with the aliphatic index of the Zyziphus spina christi, which was higher than other surfactants (Table 1). The Zyziphus spina christi, as a surfactant with a lower HLB (according to characteristic indexes in Table 1), is more efficient for accumulating the long-chain saturates in the emulsion phase. The Aliphatic/Total index of the oil phase increases in oil phases in contact with Morus nigra and Aloevera surfactants, indicating a lower affinity of the mentioned surfactants for adsorbing the long saturate chains in the emulsion. In contrast, the Zyziphus spina christi and Glycyrrhiza glabra were more efficient for accumulating long chains. The Aromatic/Total index of oil phases, in contact with xanthan and xanthan-surfactant solutions, were higher than the crude one, indicating the high potential of the aromatic hydrocarbons for accumulating in the emulsion phase. The lowest and highest Aromatic/Total index is respectively observed in the Morus nigra and Zyziphus spina christi surfactants. Based on the Polar index of the oil phases, except for the Zyziphus spina christi, the polar hydrocarbons are more involved in the emulsion phase, indicating the activation of electrostatic forces for emulsification. This observation is consistent with the polar indexes of the surfactant in Table 1, where Zyziphus spina christi has the highest Aliphatic index (0.092). The polar index of the oil phases has a synergic trend with aromatic index, representing that the aromatic hydrocarbons might attach to polar groups. Similar to the Polar index, the sulfoxide and carbonyl indexes of the oil phases were lower than the indexes in crude oil. The surfactants exhibiting higher zeta potential values demonstrated the lowest polar, carbonyl, and sulfoxide indexes, thus confirming that electrostatic forces serve as the predominant driving factor for the emulsification of surfactants derived from leaves. The effect of the xanthan polymer and surfactant concentrations on the chemistry of the oil phase (or indirectly emulsion phase) could be investigated in Tables 3, 4, 5.

**Table 2:** The FTIR characteristic indexes of the bulk oil phase, which were in contact with xanthan gum solution (2 g/L) without and with surfactants (0.2 g/L).

Indexes	Crude	Xanthan	Morus nigra	Aloevera	Zyziphus spina christi	Glycyrrhiza glabra
Aromatic/Aliphatic	0.060	0.088	0.060	0.084	0.186	0.100
Aliphatic length	1.709	1.638	1.876	1.834	1.360	1.714
Aliphatic/Total	0.273	0.268	0.316	0.290	0.187	0.267
Aromatic/Total	0.016	0.023	0.019	0.024	0.035	0.026
Polar/Total	0.023	0.019	0.020	0.020	0.038	0.026
Carbonyl/Total	0.034	0.008	0.007	0.012	0.013	0.011
Sulfoxide/Total	0.023	0.011	0.009	0.012	0.019	0.012
Condensing	0.62	0.950	0.868	1.030	1.032	0.971

**Table 3:** The FTIR characteristic indexes of the bulk oil phase, which were in contact with xanthan gum solution (2 g/L) without and with surfactants (0.5 g/L).

Indexes	Crude	Xanthan	Morus nigra	Aloevera	Zyziphus spina christi	Glycyrrhiza glabra
Aromatic/Aliphatic	0.060	0.088	0.094	0.137	0.099	0.096
Aliphatic length	1.709	1.638	1.633	1.502	1.556	1.584
Aliphatic/Total	0.273	0.268	0.262	0.218	0.250	0.255
Aromatic/Total	0.016	0.023	0.024	0.030	0.024	0.024
Polar/Total	0.023	0.019	0.024	0.030	0.024	0.027
Carbonyl/Total	0.034	0.008	0.010	0.010	0.010	0.008
Sulfoxide/Total	0.023	0.011	0.012	0.016	0.011	0.012
Condensing	0.62	0.950	0.962	0.979	0.939	0.901

**Table 4:** The FTIR characteristic indexes of the bulk oil phase, which were in contact with xanthan gum solution (5 g/L) with and without surfactants (0.2 g/L).

Indexes	Crude	Xanthan	Morus nigra	Aloevera	Zyziphus spina christi	Glycyrrhiza glabra
Aromatic/aliphatic	0.060	0.121	0.160	0.127	0.137	0.090
Aliphatic length	1.709	1.481	1.560	1.465	1.561	1.636
Aliphatic/total	0.273	0.232	0.212	0.221	0.230	0.263
Aromatic/total	0.016	0.028	0.034	0.028	0.031	0.023
Polar/total	0.023	0.024	0.020	0.022	0.019	0.022
Carbonyl/total	0.034	0.012	0.025	0.016	0.017	0.012
Sulfoxide/total	0.023	0.013	0.020	0.015	0.015	0.012
Condensing	0.624	0.946	1.19	1.026	1.013	0.965

**Table 5:** The FTIR characteristic indexes of the bulk oil phase, which were in contact with xanthan gum solution (5 g/L) without and with surfactants (0.5 g/L).

Indexes	Crude	Xanthan	Morus nigra	Aloevera	Zyziphus spina christi	Glycyrrhiza glabra
Aromatic/aliphatic	0.060	0.121	0.091	0.147	0.152	0.123
Aliphatic length	1.709	1.481	1.652	1.431	1.411	1.474
Aliphatic/total	0.273	0.232	0.269	0.215	0.206	0.228
Aromatic/total	0.016	0.028	0.024	0.031	0.031	0.028
Polar/total	0.023	0.024	0.020	0.027	0.025	0.023
Carbonyl/total	0.034	0.012	0.010	0.012	0.013	0.018
Sulfoxide/total	0.023	0.013	0.010	0.015	0.017	0.014
Condensing	0.624	0.946	0.878	0.984	1.038	1.035

Table 3 summarizes the FTIR indexes of oil phases in contact with xanthan solution bearing a high concentration of surfactants. As shown in the Aromatic/Aliphatic index, increasing the surfactant concentration leads to more accumulation of aromatic hydrocarbons in the emulsion phase of Zyziphus spina christi (0.099). By increasing the surfactant concentration (Morus nigra, Aloevera, and Glycyrrhiza glabra), the length of the saturated hydrocarbons in the bulk phase increases (Zyziphus spina christi surfactant showed an inverse trend). This observation is consistent with the Aliphatic/Total index, which is reduced in the Morus nigra (0.262), Aloevera (0.218), and Glycyrrhiza glabra (0.255) emulsions. The Aromatic/Total indexes followed diverse trends in the bulk oil phases. The Polar/Total index of the oil phases, which were in contact with the Morus nigra (0.024), Aloevera (0.030), and Glycyrrhiza glabra (0.027), increased by increasing surfactant concentration. In contrast, according to the Polar/Total index of the oil phase in contact with the Zyziphus spina christi surfactant, the polarity reduces by increasing the surfactant concentration. The increase in the surfactant concentration did not lead to a considerable change in the carbonyl index. The increase in the concentration of Zyziphus spina christi surfactant, as compared to other factors, resulted in a decrease in the sulfoxide index, indicating a greater presence of sulfuric compounds in the emulsion phase at higher concentrations. Diverse condensing indexes were observed by increasing each surfactant concentration. For instance, despite other surfactants, the condensing index of the oil phase, which was in contact with Morus nigra, was increased by increasing the surfactant concentration.

Table 4 reports the characteristic indexes obtained from emulsions generated by xanthan solutions with a concentration of 5 g/L. By comparing the FTIR indexes of 2 g/L and 5 g/L xanthan emulsions, the effect of xanthan concentration on the oil bulk chemistry could be investigated. The affinity of aromatic and aliphatic hydrocarbons toward the emulsion phase reduces and increases, respectively. The length of the aliphatic hydrocarbon was reduced as a result of the xanthan concentration increasing. Besides, by increasing the xanthan concentration, the affinity of alcoholic, amine, and carbonyl-bearing hydrocarbons toward the emulsion phase reduces. The condensing index, which represents the aromatic fusion degree, did not change by increasing concentration. The alteration of the Aromatic/Aliphatic index of oil phases in contact with the xanthan solutions (5 g/L) bearing surfactants (0.2 g/L) changed by the surfactant chemistry. For instance, oil phases in contact with Morus nigra (0.160) and Aloevera (0.127) had a higher Aromatic/Aliphatic index, while the other did not. Regarding the aliphatic and aromatic indexes, increasing the xanthan concentration led to more contribution of aliphatic compounds in the emulsion phase. Despite other surfactants, the Zyziphus spina christi could lower the polar index (0.019) at a higher xanthan concentration. Increasing the xanthan concentration reduced the affinity of carbonyl and sulfoxide-bearing hydrocarbons toward the emulsion phase. Except for the Morus nigra, increasing the xanthan concentration did not lead to a noticeable change in the condensing index of bulk oil.

Table 5 shows the FTIR index of the oil phases with xanthan-surfactant solutions with high concentrations. The Aromatic/Aliphatic index of oil phases in contact with xanthan-surfactant solutions with high concentrations was generally higher than solutions with lower concentrations. The Aromatic index of the oil phase in contact with Glycyrrhiza glabra and Aloe vera (with high surfactant and xanthan concentration) exhibited an increase, suggesting a reduced affinity of aromatic hydrocarbons towards the emulsion phase with the rising concentrations of surfactant and polymer. The highest carbonyl index was observed in the oil phase in contact with the xanthan and Glycyrrhiza glabra with high concentration. Similar to the carbonyl index, the condensing index of the oil phase in contact with Glycyrrhiza glabra increased with polymer and surfactant concentration. According to the characteristic indexes, given by the FTIR technique, the chemistry, and as a result, the type of emulsion generated by a xanthan-bio-based surfactant is a function of surfactant chemistry, as well as polymer and surfactant concentrations.

### Asphaltene molecular characterization

**Figure 6:**
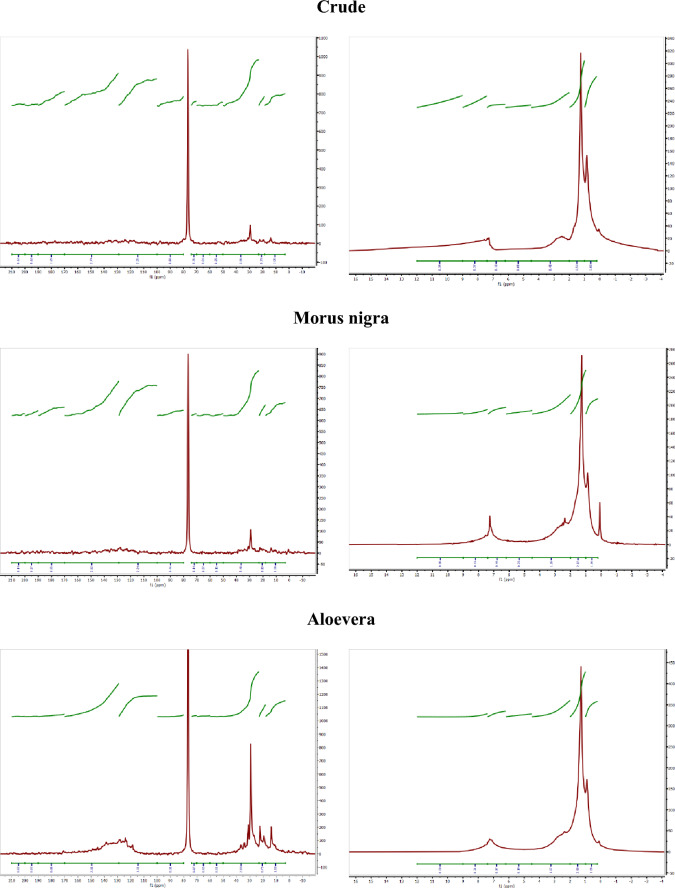

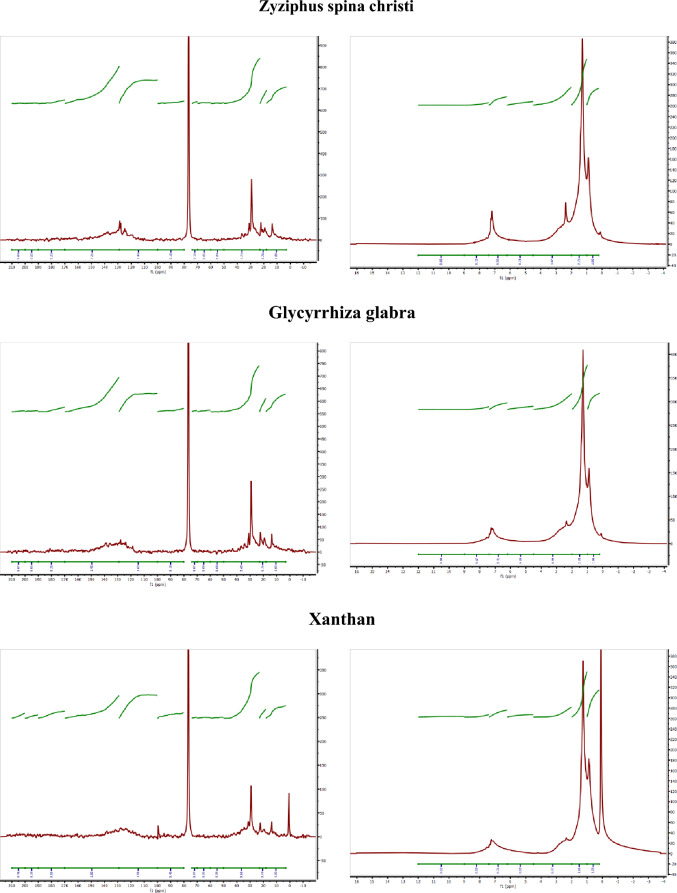
The HNMR and CNMR spectra of the crude and bulk asphaltenes in contact with the xanthan solution (5 g/L) without and with surfactants (0.5 g/L).

To examine the structural characteristics of asphaltenes present in the emulsion phase, the NMR spectroscopy technique was conducted on the asphaltenes within the bulk phase. Figure 6 shows the NMR (HNMR and CNMR) spectra of the crude asphaltene, and the bulk ones, which were in contact with polymer-surfactant solutions.

The peaks in HNMR and CNMR spectra, which represent a covalent bond of hydrogen and carbon atoms, confirm the presence of aliphatic and aromatic compounds bearing various functional groups. Based on the observed chemical shifts, it can be deduced that asphaltene molecules consist of fused aromatic rings linked to aliphatic chains. The presence of quaternary aromatic carbon in CNMR spectra confirms the presence of fused aromatic rings, suggesting the island model as the probable molecular model for asphaltene. The presence of associated aliphatic chains could be captured by corresponding ranges in the CNMR and HNMR spectra. The functional groups, such as carboxylic acids, aldehydes, alcoholics, amines, and ether are captured in the spectra^31,43–51^. More detailed information about shift assignments of the HNMR and CNMR spectra can be found in Tables A1, A2 in the appendix. The structural parameters, shown as Eqs. 1–4, are used to determine the asphaltene molecular structure.

1 $$n=\frac{{H}_{(0.1-1)}+{H}_{(1-2)}+{H}_{(2-4.5)} }{{H}_{\left(2-4.5\right) }}$$

2 $$\varphi =\frac{{C}_{p}}{{C}_{ar}}$$

3 $${f}_{a}=\frac{{C}_{ar}}{{C}_{ar}+{C}_{al}}$$

4 $${I}_{C=O}=\frac{{H}_{(9-12)}}{Total area}$$

In Eq. 2–3, each of the "C" designations corresponds to specific integral areas beneath the CNMR spectrum within defined ranges. "C_ar_" and "C_al_" represent carbons in aromatic rings and aliphatic chains, respectively, determined by their integral areas in the aromatic and aliphatic ranges (Tables A1, A2 in the appendix). "C_p_" refers to peripheral aromatic carbons situated on the edges of aromatic cores, excluding internal ones. The ratio of peripheral carbons (C_p_) to total aromatic carbons (C_ar_) is directly proportional to the size of the aromatic core or the number of fused aromatic rings, providing an approximation of the aromatic fused ring number. This ratio, known as the shape factor (φ), is calculated as C_p_/C_ar_. Furthermore, aromaticity (f_a_) is demonstrated by the ratio of aromatic carbons (C_ar_) to the sum of aromatic and aliphatic carbons (C_ar_ + C_al_). The integral areas (C_ar_, C_al_, and C_p_) are dimensionless, along with the aromaticity, shape factor, and ${I}_{C=O}$.

In Eq. 1, which is derived from HNMR spectra, ‘H_x_ ‘, ‘Total area’, and ‘n’ stand for the normalized integral area in the range ‘x’, total area, and aliphatic chain length in terms of carbon number, respectively. The dimension of ‘n’ is the number of carbon in an aliphatic chain. In addition, in Eq. 4, ‘H_(9–12)_‘ and ‘Total area’ stand for the normalized integral area of the HNMR spectrum in the ranges of 9–12 ppm and total range, respectively.

The integral areas of the HNMR and CNMR, which are used to calculate the indexes, were obtained using the MestReNova software package. Aside from the integral area calculation, the MestReNova was employed to process the NMR spectra for denoising, apodization, baseline correction, and phase correction^31^.

The structural characteristics of asphaltenes within both the crude and bulk samples are presented in Table 6. As shown in Table 6, the number of the fused aromatic rings from 9 in crude asphaltene reduced to 7 in the bulk asphaltene in contact with the xanthan solution (without surfactant). The number of fused rings aromatic increased to 10–11 in the bulk asphaltenes in contact with Aloevera, Zyziphus spina christi, and Glycyrrhiza glabra. In addition, the number of fused aromatic rings in bulk asphaltene in contact with Morus nigra surfactant was equal to the crude one. The changes in fused aromatic ring number, which was determined according to the shape factor, were consistent with the zeta potential measurements. The xanthan solution (without surfactant) had the highest zeta potential value, representing more potential for establishing electrostatic interaction with asphaltene molecules. Besides, the xanthan solution bearing Morus nigra surfactant had a higher zeta potential value among the surfactant solutions. Regarding the number of aromatic rings in the bulk asphaltene and zeta potential measurements, it could be concluded that increasing the polarity of the surfactant leads to the accumulation of larger asphaltenes in the emulsion phase. The lower aromaticity of the bulk asphaltene in contact with the xanthan solution (without surfactant) is consistent with the fused aromatic ring number. The aliphatic chain length of the bulk asphaltene changed according to the chemistry of the xanthan-surfactant solution. While the aliphatic chain length of the crude asphaltene is 6.22 carbons, the chain length has increased to 8.18 carbons in the bulk asphaltene in contact with the xanthan solution (without surfactant). The addition of surfactants to the xanthan solution reduced the asphaltene aliphatic chain length to the range of 4.07–4.81 carbons. Hence, the surfactants have the potential to adsorb the asphaltenes with longer chains in the emulsion phase. As indicated by the I_c=o_ index, compounds containing carbonyl groups exhibit lower affinities for adsorption into the emulsion phase formed by the xanthan solution (in the absence of surfactant). The I_c=o_ index of the xanthan-surfactant solution bearing Aloevera and Zyziphus spina christi-derived surfactant was lower than others, representing more tendency of carbonyl-rich asphaltenes toward emulsion. The changes in the I_c=o_ index are also consistent with zeta potential values. The compounds with more negative zeta potential values have a lower affinity to adsorb the asphaltenes-bearing carbonyl-based functional groups. The high electron density of the C=O group could repel the negatively charged molecules, leading to a lower contribution of carbonyl-rich asphaltenes in the emulsion phase. Analysis of asphaltene structural parameters obtained through NMR indicates that highly aliphatic asphaltenes with a smaller aromatic core exhibit greater affinity for contributing to the emulsion phase when using bio-based surfactants. This suggests that oil samples containing asphaltenes with these molecular characteristics possess increased potential for emulsion generation when injected with a solution of bio-based surfactant and xanthan polymer. Consequently, the process of emulsification, a fluid–fluid mechanism, is significantly influenced by asphaltene structure.

**Table 6:** The structural parameters of the crude and bulk asphaltenes in contact with the xanthan solution (5 g/L) without and with surfactants (0.5 g/L).

Parameter	Crude	Xanthan	Morus nigra	Aloevera	Zyziphus spina christi	Glycyrrhiza glabra
Car	4.99	3.61	4.86	3.42	3.67	2.98
Cp	2.25	1.81	2.24	1.31	1.41	1.03
Cal	5.94	5.47	5.38	4.6	4.53	4.37
$\varphi$	0.45	0.50	0.46	0.38	0.38	0.34
Fused ring number	9	7	9	10	10	11
f_a_	0.45	0.39	0.47	0.42	0.44	0.40
Aliphatic chain length	6.22	8.18	4.07	4.64	4.47	4.81
I_c=o_	0.008	0.037	0.011	0.002	0.001	0.008

### Bulk oil viscosity

The viscosity measurement was used to address the mechanical properties of the bulk oil. Regarding the fact that the chemistry of the bulk oil and asphaltene structure has changed after the emulsification, the alteration of bulk oil viscosity was expected. Table 7 shows the viscosity of the bulk oil, which was in contact with xanthan solution (without surfactant) and surfactant-bearing xanthan solution at various concentrations.

**Table 7:** The viscosity of the bulk oil, which was in contact with A. xanthan (2 g/L)-surfactant (0.2 g/L), B. xanthan (2 g/L)-surfactant (0.5 g/L), C. xanthan (5 g/L)-surfactant (0.2 g/L), and xanthan (5 g/L)-surfactant (0.5 g/L).

Viscosity (cP)/ Aqueous phase	Xanthan	Morus nigra	Aloevera	Zyziphus spina christi	Glycyrrhiza glabra
Polymer (2 g/L) Surfactant (0.2 g/L)	42.1	42.4	29.5	26	35
Polymer (2 g/L) Surfactant (0.5 g/L)		31.2	35	42.8	26.2
Polymer (5 g/L) Surfactant (0.2 g/L)	29.7	35.7	27.7	38.6	41.8
Polymer (5 g/L) Surfactant (0.5 g/L)		39.5	51.9	39.9	42

*The repeatability of viscometric tests was $\pm$ 0.3 cP.

The rheological behavior of the oil sample could be expressed by Eq. 5. In this equation, colloidal dispersion viscosity (${\mu }_{r})$ could be found by dividing colloidal dispersion viscosity ($\mu )$ by the dispersing liquid one (${\mu }_{0})$. The variables of ${\phi }_{eff}$, ${k}_{12}$, and $\upsilon$ indicate the effective volume fraction after the solvation, coefficient, and shape factor, respectively. In addition, the variable ‘K’, which is called the solvation constant, is obtained through the division of the effective volume fraction by the original dry volume fraction ($\phi$). Considering $\left[\mu \right]=K\upsilon$ and ${\phi }_{eff}=K \phi$, Eq. 5 could be rewritten as Eq. 6, as follows^52,53^ :

5 $${\mu }_{r}=\frac{\mu }{{\mu }_{0}}=(1+\upsilon {\phi }_{eff}+{k}_{1}{\phi }_{eff}^{2}+{k}_{2}{\phi }_{eff}^{3}+\dots )$$

6 $$\mu ={\mu }_{0}(1+\left[\mu \right]\phi +{K}_{1}{\phi }^{2}+{K}_{2}{\phi }^{3}+\dots )$$

Concerning Eqs. 5–6, an increase in the proportion of larger dispersed particles, such as asphaltenes and waxes, results in higher oil viscosity, whereas the presence of lighter hydrocarbons within the bulk oil reduces viscosity. Therefore, modifications to the oil's bulk viscosity could potentially mitigate variations in the relative concentrations of dispersed asphaltenes and dispersing hydrocarbons.

As shown in Fig. 6, the viscosity of bulk oil samples changes with the chemistry and concentration of the xanthan-surfactant solution. The general trend of viscosity alterations was unique for each surfactant. The viscosity of the bulk oil, when exposed to the xanthan solution (without surfactant), decreased with the increasing concentration of xanthan. This means that increasing xanthan concentration enhances the large hydrocarbon participation in the emulsion phase, leading to bulk oil viscosity reduction. The viscosity alteration was more complicated at the xanthan solution-bearing surfactant. The viscosity of the bulk oil in contact with the xanthan solution containing Morus nigra does not exhibit a discernible pattern or trend. The increasing Morus nigra could either reduce or increase the bulk phase viscosity. The viscosity of the bulk oil phases, when in contact with surfactants derived from Aloevera and xanthan polymer at varying concentrations, exceeded that of other samples. This issue shows increasing Aloevera-derived surfactant and xanthan concentrations could accumulate the light hydrocarbons in the emulsion phase, leading to an increase in the bulk oil viscosity. By increasing the Zyziphus spina Christi and xanthan concentration, the viscosity of the bulk oil increases as a result of adsorbing the light hydrocarbons in the emulsion phase. In the emulsions produced using Glycyrrhiza glabra, the bulk oil viscosity was observed to increase when treated with solutions containing higher concentrations of xanthan.

### Microfluidic injection studies

**Figure 7:**
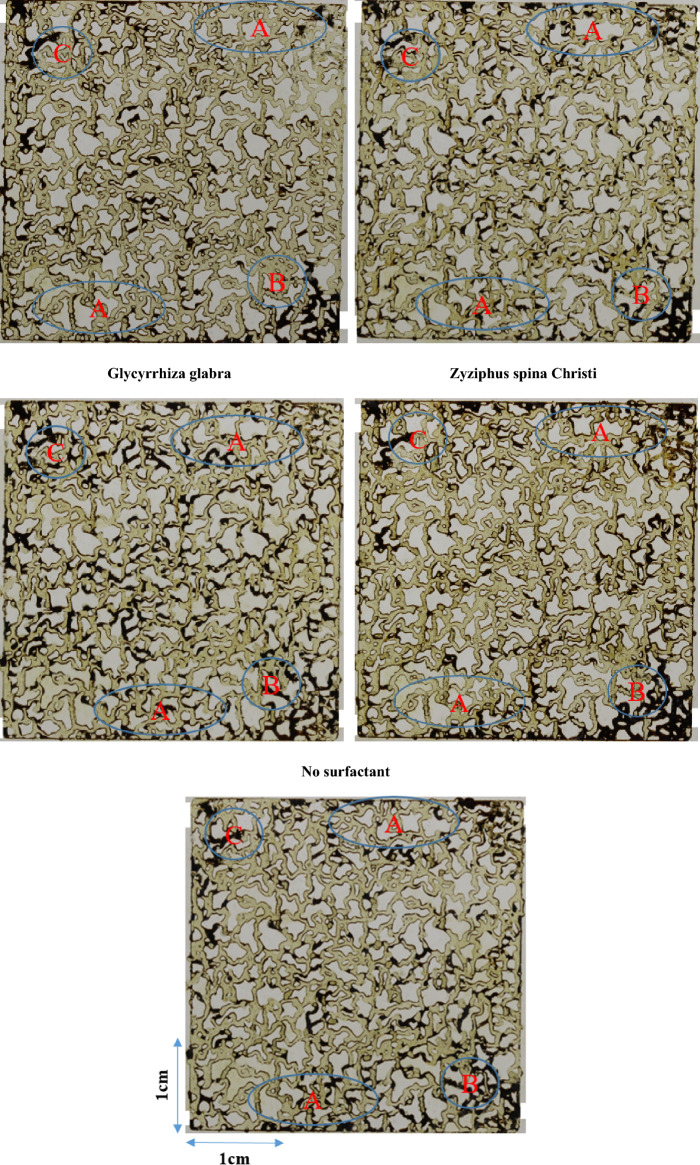
The ultimate micromodel image was captured at the end of the xanthan solution and xanthan-surfactant injection.

To investigate fluid–fluid behavior dynamically, microfluidic injection was employed using solutions containing xanthan and surfactant at concentrations of 2 g/L and 0.5 g/L, respectively. Besides, similar to the previous experiments, xanthan solution without surfactant was also studied to find the effect of surfactants without the presence of xanthan polymer. Figure 7 illustrates the final images of the micromodel after oil production ceased due to microfluidic injections. In Region A of the images, oil displacement appears more piston-like with Morus nigra and Aloevera surfactant-xanthan injections, indicating lower capillary pressure within the micromodel. Capillary pressure is influenced by interfacial tension and wettability conditions (contact angle). Given that the wettability alteration mechanism is inactive during short-term microfluidic injections, the reduced capillary pressure should be caused by lower interfacial tension. Moreover, in areas where fluid flow is radial—near production (Region B) and injection spots (Region C)—the sweep efficiency of bio-based surfactant-rich solutions is lower compared to xanthan solutions without surfactant.

**Figure 8:**
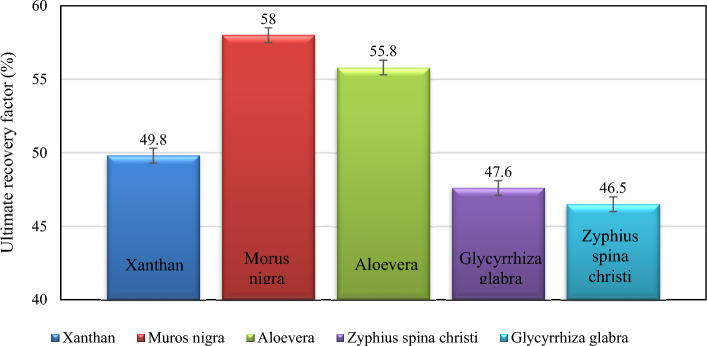
The ultimate recovery factor resulted from the injection of xanthan and xanthan-leaf-derived surfactants, with a repeatability of ± 0.5%.

Figure 8 illustrates the final oil recovery outcomes following the injection of different xanthan and xanthan-surfactant solutions. The highest and lowest oil recoveries were observed with Xanthan-Morus nigra (58.0%) and Xanthan-Glycyrrhiza glabra (46.5%) solutions, respectively. Among the leaf-derived surfactants tested, the addition of Morus nigra and Aloevera-derived surfactants improved oil recovery, whereas Zyphius spina christi and Glycyrrhiza glabra led to decreased oil recovery. The microfluidic injections provided insights into dynamic fluid–fluid interactions. Based on these injections, incorporating Morus nigra and Aloevera-derived surfactants appears promising for enhancing oil recovery. Consequently, composite polymer-surfactant solutions such as Xanthan-Morus nigra-derived and Xanthan-Aloevera-derived surfactants are highly recommended for this purpose.

The engineering application can be evaluated from three perspectives: feasibility of bio-surfactant extraction, economic viability, and environmental impact. Our laboratory findings, in alignment with existing literature, revealed that bio-surfactants extracted from leaf waste amounted to 9–17% by weight of the initial waste^54,55^. To put this into context, the leaf waste from just 100 trees can produce nearly 4000 gallons of injectable fluid (at a concentration of 0.5 g/L), a substantial quantity. While our study utilized a Soxhlet apparatus for extraction at the lab scale, larger industrial extractors capable of processing 1000 kg/hr exist for field-scale applications. Furthermore, the economic feasibility of these bio-surfactants is notable, priced at approximately $2–3 per kilogram due to their extraction from waste leaves. Additionally, given their natural origin, the use of these bio-surfactants is anticipated to have no adverse environmental impacts.

## Conclusion

This research investigated the fluid–fluid interaction of leaf-derived surfactant-xanthan gum as a novel green polymer-surfactant composite fluid for improving oil recovery. The surfactants were extracted from Morus nigra, Aloevera leaf, Zyziphus spina christi, and Glycyrrhiza glabra leaves. The following statements were concluded from surfactant characterization tests and conducted experiments.

1. The FTIR, zeta potential measurement, DLS, and CMC calculation were conducted to characterize the leaf-derived surfactants. As indicated in the characteristic indexes, obtained from the FTIR spectra, more electrically polar functional groups, such as carbonyl and halide, are higher in Aloevera and Morus nigra surfactants, contributing to the hydrophilicity of the surfactants. On another hand, the higher aliphatic indexes of the Zyziphus spina christi and Glycyrrhiza glabra-derived surfactants indicated a higher hydrophobicity for these surfactants. The xanthan solution (without surfactant) had a higher zeta potential value, representing higher electrostatic force. The addition of leaf-derived surfactant led to a reduction of zeta potential value as a sign of compensation of the electrostatic force. According to the DLS spectra, the surfactant aggregates are smaller in Glycyrrhiza glabra and Aloevera solutions. The CMC of the surfactants was in the range of 14.74–33.39 g/L, and the lowest CMC was observed in the Aloevera-derived surfactant.
2. FTIR spectroscopy was employed to investigate alterations in the bulk oil phase following emulsification. The Aromatic/Aliphatic indexes in the bulk oil phase were consistently higher compared to those in crude oil, suggesting a greater affinity of aliphatic hydrocarbons for participation in the emulsion phase. Based on the FTIR condensing index, in comparison to fused aromatic hydrocarbons, the lighter aromatic hydrocarbons have a greater contribution to the emulsion phase. According to the condensing and Aromatic/Aliphatic indexes, the Zyziphus spina christi and Morus nigra solutions had lower and higher interaction with aromatic hydrocarbons, respectively. On the other hand, the long-chain saturates had more affinity toward the emulsion phase generated by the Zyziphus spina christi solution. The lowest carbonyl, polar, and sulfoxide indexes were observed in the surfactants with higher zeta potential values, confirming that electrostatic forces serve as the primary driving factor behind the emulsification process.
3. According to the structural parameters obtained from NMR analysis of the bulk oil phase, it is evident that the xanthan solution has the capacity to accumulate asphaltenes characterized by larger aromatic cores. The addition of surfactant into the xanthan reduces the solution's ability to accumulate the highly fused aromatic asphaltenes. In contrast, the addition of surfactant to the xanthan solution improved its ability to adsorb asphaltenes with longer associate chains. The I_c=o_ index complied with zeta potential values. The compounds with more negative zeta potential values had a lower tendency to adsorb the asphaltenes-bearing carbonyl-based functional groups. The high electron density of the C=O group repelled the negatively charged hydrocarbons, resulting in lower participation of carbonyl-rich asphaltenes in the emulsion phase.
4. The bulk oil viscosity was used to address the contents of large dispersed hydrocarbons and dispersing bulk hydrocarbons. The viscosity of the bulk oil, upon exposure to the xanthan solution (without surfactant), diminished as the xanthan concentration increased. The viscosity of solutions bearing surfactant had a diverse trend in response to increasing polymer and surfactant concentrations.
5. The microfluidic injections were employed to investigate the xanthan-leaf-derived surfactant from a dynamic point of view. Based on the microfluidic injections, the presence of Morus nigra and Aloevera leaf-derived surfactants improved ultimate oil recovery. While injection of xanthan solution leads to recovery of 49.84% of the original oil, the addition of Morus nigra and Aloevera leaf-derived surfactants increased the oil recovery to 58.06 and 55.84%, respectively. Regarding the fact that the investigated polymer-surfactant composite is entirely based on the abundant materials in nature, it is highly recommended from environmental and economic aspects.

### Supplementary Information

Supplementary Information.

## Data Availability

More detailed data will be provided by contacting H.V. (vatanparasth@ripi.ir).

## References

[1] Salehi N, Saeedi Dehaghani A, Haghighi M (2023). Investigation of fluid-fluid interaction between surfactant-ion-tuned water and crude oil: A new insight into asphaltene behavior in the emulsion interface. J. Mol. Liq..

[2] Nourani, M. & Sadeghnejad, S. Alkaline-surfactant polymer (ASP). in *Chemical Methods* 821931 (2022).

[3] Tajikmansori A, Hosseini M, Dehaghani AHS (2021). Mechanistic study to investigate the injection of surfactant assisted smart water in carbonate rocks for enhanced oil recovery: An experimental approach. J. Mol. Liq..

[4] Kalam S (2023). Static and dynamic adsorption of a gemini surfactant on a carbonate rock in the presence of low salinity water. Sci. Rep..

[5] Noorizadeh Bajgirani SS, Saeedi Dehaghani AH (2023). Experimental investigation of wettability alteration, IFT reduction, and injection schemes during surfactant/smart water flooding for EOR application. Sci. Rep..

[6] Dehaghani AHS, Hosseini M, Tajikmansori A, Moradi H (2020). A mechanistic investigation of the effect of ion-tuned water injection in the presence of cationic surfactant in carbonate rocks: An experimental study. J. Mol. Liq..

[7] Agi A, Junin R, Gbonhinbor J, Onyekonwu M (2018). Natural polymer flow behaviour in porous media for enhanced oil recovery applications: A review. J. Pet. Explor. Prod. Technol..

[8] Ramos de Souza E (2022). Xanthan gum produced by Xanthomonas campestris using produced water and crude glycerin as an environmentally friendlier agent to enhance oil recovery. Fuel.

[9] Soliman AA, El-Hoshoudy AN, Attia AM (2020). Assessment of xanthan gum and xanthan-g-silica derivatives as chemical flooding agents and rock wettability modifiers. Oil Gas Sci. Technol..

[10] Said M (2021). Modification of xanthan gum for a high-temperature and high-salinity reservoir. Polymers (Basel).

[11] Sowunmi A, Efeovbokhan VE, Orodu OD, Olabode O, Oputa A (2022). Comparative study of biopolymer flooding: A core flooding and numerical reservoir simulator validation analysis. Model. Simul. Eng..

[12] Fu X, Qin F, Liu T, Zhang X (2022). Enhanced oil recovery performance and solution properties of hydrophobic associative Xanthan Gum. Energy and Fuels.

[13] Nazarahari MJ (2021). Impact of a novel biosynthesized nanocomposite (SiO2@Montmorilant@Xanthan) on wettability shift and interfacial tension: Applications for enhanced oil recovery. Fuel.

[14] Xu L (2023). Temperature/salt tolerance and oil recovery of xanthan gum solution enhanced by surface-modified nanosilicas. Pet. Sci..

[15] Ji Z (2023). Molecular dynamics simulation of CO2 storage in reservoir pores with a dead-end. Energies.

[16] Machale J, Majumder SK, Ghosh P, Sen TK (2019). Development of a novel biosurfactant for enhanced oil recovery and its influence on the rheological properties of polymer. Fuel.

[17] Navaie F, Esmaeilnezhad E, Jin Choi H (2022). Xanthan gum-added natural surfactant solution of Chuback: A green and clean technique for enhanced oil recovery. J. Mol. Liq..

[18] Saha R, Uppaluri RVS, Tiwari P (2019). Impact of natural surfactant (Reetha), polymer (Xanthan Gum), and silica nanoparticles to enhance heavy crude oil recovery. Energy Fuels.

[19] Ahmadi MA, Shadizadeh SR (2018). Spotlight on the New Natural Surfactant Flooding in Carbonate Rock Samples in Low Salinity Condition. Sci. Rep..

[20] Emadi S, Shadizadeh SR, Manshad AK, Rahimi AM, Mohammadi AH (2017). Effect of nano silica particles on Interfacial Tension (IFT) and mobility control of natural surfactant (Cedr Extraction) solution in enhanced oil recovery process with nano - surfactant flooding. J. Mol. Liq..

[21] Esfandyari H, Shadizadeh SR, Esmaeilzadeh F, Davarpanah A (2020). Implications of anionic and natural surfactants to measure wettability alteration in EOR processes. Fuel.

[22] Emadi S (2019). Effect of using Zyziphus Spina Christi or Cedr Extract ( CE ) as a natural surfactant on oil mobility control by foam fl ooding. J. Mol. Liq..

[23] Moslemizadeh A, Shirmardi Dezaki A, Shadizadeh SR (2017). Mechanistic understanding of chemical flooding in swelling porous media using a bio-based nonionic surfactant. J. Mol. Liq..

[24] Shadizadeh SR, Moslemizadeh A, Dezaki AS (2015). A novel nonionic surfactant for inhibiting shale hydration. Appl. Clay Sci..

[25] Datta P, Tiwari P, Pandey LM (2022). Experimental investigation on suitability of Surfactin for enhanced oil recovery: Stability, adsorption equilibrium and kinetics studies. J. Environ. Chem. Eng..

[26] Razzaghi-Koolaee F, Mehrabianfar P, Soltani Soulgani B, Esfandiarian A (2022). A comprehensive study on the application of a natural plant-based surfactant as a chemical enhanced oil recovery (CEOR) agent in the presence of different ions in carbonate reservoirs. J. Environ. Chem. Eng..

[27] Daghbandan A, Shahrabadi A, Arabiyoun M (2022). Adsorption of Glycyrrhiza glabra natural nonionic surfactant onto the carbonate reservoir rock in the presence of SiO2 nanoparticles surface: Towards enhanced oil recovery. J. Environ. Chem. Eng..

[28] Shi J (2004). Saponins from edible legumes: Chemistry, processing, and health benefits. J. Med. Food.

[29] Schmitt C (2014). Saponins: A renewable and biodegradable surfactant from its microwave-assisted extraction to the synthesis of monodisperse lattices. Biomacromolecules.

[30] Ruiz-Morales Y, Miranda-Olvera AD, Portales-Martĺnez B, Domĺnguez JM (2020). Experimental and theoretical approach to determine the average asphaltene structure of a crude oil from the golden lane (Faja de Oro) of Mexico. Energy Fuels.

[31] Karami S, Hossein A, Dehaghani S (2022). A molecular insight into cracking of the asphaltene hydrocarbons by using microwave radiation in the presence of the nanoparticles acting as catalyst. J. Mol. Liq..

[32] Luan Y (2022). Effect of the water film rupture on the oil displacement by supercritical CO2 in the nanopore: Molecular dynamics simulations. Energy Fuels.

[33] Amir Hosseini M, Kamrava S, Sahimi M, Tahmasebi P (2023). Effect of wettability on two-phase flow through granular porous media: Fluid rupture and mechanics of the media. Chem. Eng. Sci..

[34] Vinarov Z, Radeva D, Katev V, Tcholakova S, Denkov N (2018). Solubilisation of hydrophobic drugs by saponins. Indian J. Pharm. Sci..

[35] Emadi S (2019). Effect of using Zyziphus Spina Christi or Cedr Extract (CE) as a natural surfactant on oil mobility control by foam flooding. J. Mol. Liq..

[36] Karami S, Saeedi Dehaghani AH, Haghighi M (2021). Investigation of smart water imbibition assisted with microwave radiation as a novel hybrid method of enhanced oil recovery. J. Mol. Liq..

[37] Karami S, Saeedi Dehaghani AH, Hossein Seyed Mousavi SA (2020). Condensate blockage removal using microwave and ultrasonic waves: Discussion on rock mechanical and electrical properties. J. Pet. Sci. Eng..

[38] Tajikmansori A, Hossein Saeedi Dehaghani A, Sadeghnejad S, Haghighi M (2023). New insights into effect of the electrostatic properties on the interfacial behavior of asphaltene and resin: An experimental study of molecular structure. J. Mol. Liq..

[39] Hemmati-sarapardeh A, Dabir B (2018). Toward mechanistic understanding of asphaltene aggregation behavior in toluene: The roles of asphaltene structure, aging time, temperature, and ultrasonic radiation. J. Mol. Liq..

[40] Zojaji I, Esfandiarian A, Taheri-Shakib J (2021). Toward molecular characterization of asphaltene from different origins under different conditions by means of FT-IR spectroscopy. Adv. Colloid Interface Sci..

[41] Karami S, Saeedi Dehaghani A, Haghighi M (2024). Analytical investigation of asphaltene cracking due to microwave and ultrasonic radiations: A molecular insight into asphaltene characterization and rheology. Geoenergy Sci. Eng..

[42] Tanhaei, H., Hossein, A., Dehaghani, S. & Karami, S. *Investigation of Microwave Radiation in Conjugate with Acidizing as a Novel Hybrid Method of Oil Well Stimulation*. 1–34.

[43] Speight JG (1970). A structural investigation of the constituents of Athabasca bitumen by proton magnetic resonance spectroscopy. Fuel.

[44] Sheremata JM, Gray MR, Dettman HD, McCaffrey WC (2004). Quantitative molecular representation and sequential optimization of Athabasca asphaltenes. Energy Fuels.

[45] Joonaki E, Buckman J, Burgass R, Tohidi B (2018). Exploration of the difference in molecular structure of n -C7 and CO2 induced asphaltenes. Ind. Eng. Chem. Res..

[46] Scotti R, Montanari L (1998). Molecular structure and intermolecular interaction of asphaltenes by FT-IR, NMR, EPR. Struct. Dyn. Asphaltenes.

[47] Daaou M (2008). Characterization of the nonstable fraction of Hassi-Messaoud asphaltenes. Energy and Fuels.

[48] Buenrostro-Gonzalez E, Andersen SI, Garcia-Martinez JA, Lira-Galeana C (2002). Solubility/molecular structure relationships of asphaltenes in polar and nonpolar media. Energy Fuels.

[49] Molina D, Ariza E, Poveda JC (2017). Structural differences among the asphaltenes in Colombian light crudes from the Colorado Oil Field. Energy Fuels.

[50] Taherian Z, Dehaghani AHS, Ayatollahi S, Kharrat R (2021). A new insight to the assessment of asphaltene characterization by using fortier transformed infrared spectroscopy. J. Pet. Sci. Eng..

[51] Yazdani B, Saeedi Dehaghani AH, Karami S (2023). Fundamental study of asphaltene cracking and desorption from rock surface due to microwave radiation: A molecular insight into catalytic effect of minerals and asphaltene chemistry. Geoenergy Sci. Eng..

[52] Mozaffari S, Tchoukov P, Atias J, Czarnecki J, Nazemifard N (2015). Effect of asphaltene aggregation on rheological properties of diluted athabasca bitumen. Energy and Fuels.

[53] Luo P, Gu Y (2007). Effects of asphaltene content on the heavy oil viscosity at different temperatures. Fuel.

[54] Yu XL, He Y (2018). Tea saponins: Effective natural surfactants beneficial for soil remediation, from preparation to application. RSC Adv..

[55] Olorunnisola O, Adetutu A, Fadahunsi O (2017). Anti-allergy potential and possible modes of action of Sphenocentrum jollyanum pierre fruit extract. J. Phytopharm..

