# Supplementary material for: New molecular and macroscopic understandings of novel green chemicals based on Xanthan Gum and bio-surfactants for enhanced oil recovery
Source: Sci Rep. 2024 Jun 3;14:12752. doi: 10.1038/s41598-024-63244-z (PMC11148032; doi:10.1038/s41598-024-63244-z)
Supplement: Supplementary file 1 — Supplementary Information. [file 41598_2024_63244_MOESM1_ESM.docx]

**Appendix**

Table A1. The chemical shift assignments in HNMR spectrums of the crude and the bulk oil phase asphaltene ^29,36–44^.

| Chemical shifts (ppm) | Assignments |
| --- | --- |
| 0.1-1 | Hydrogens in aliphatic chains (γ to the aromatic ring, CH_3_ groups) |
| 1-2 | Hydrogens in aliphatic chains (β to aromatics, CH_3_, and CH_2_) |
| 2-4.5 | CH _(1-3)_ and α to aromatics. |
| 4.5-6.2 | Olefin hydrogen in CH_(1&2)_ |
| 6.2-9 | Aromatic hydrogens |
| 9-12 | Hydrogens in Aldehydic and carboxylic groups |

Table A2. The chemical shift assignments in CNMR spectrums of the crude and the bulk oil phase asphaltene ^29,36–44^.

| Chemical shifts (ppm) | Assignments |
| --- | --- |
| 3-18.5 | CH_3_ group located at the end of aliphatic chains |
| 18.5-23 | Carbons with methyl groups situated in the α position relative to aromatic rings. |
| 23-50 | Carbons at positions CH(1-2) within naphthenic and paraffinic compounds |
| 50-60 | CH (0-1) in paraffinic carbons, C-N (representing amine groups), carbons attached to sulfur |
| 60-76 | Carbon atoms in paraffinic and naphthenic compounds positioned α to the OH group |
| 78-100 | Ether, ester, and alcohol |
| 100-129 | Aromatic carbon located on the periphery |
| 129-170 | Aromatic carbon in the quaternary position |
| 170-190 | C=O located in Carboxylic acid |
| 190-200 | Carbons within the quinoline structure |
| >200 | C=O within aldehyde and ketone groups |
